# Printed peptide arrays identify prognostic TNC serumantibodies in glioblastoma patients

**DOI:** 10.18632/oncotarget.3791

**Published:** 2015-04-12

**Authors:** Andreas Mock, Rolf Warta, Christoph Geisenberger, Ralf Bischoff, Alexander Schulte, Katrin Lamszus, Volker Stadler, Thomas Felgenhauer, Christian Schichor, Christoph Schwartz, Jakob Matschke, Christine Jungk, Rezvan Ahmadi, Felix Sahm, David Capper, Rainer Glass, Jörg-Christian Tonn, Manfred Westphal, Andreas von Deimling, Andreas Unterberg, Justo Lorenzo Bermejo, Christel Herold-Mende

**Affiliations:** ^1^ Department of Neurosurgery, Experimental Neurosurgery, University of Heidelberg, Heidelberg, Germany; ^2^ PEPperPRINT GmbH, Heidelberg, Germany; ^3^ Division of Functional Genome Analysis, German Cancer Research Centre (DKFZ), Heidelberg, Germany; ^4^ Department of Neurosurgery, Laboratory for Brain Tumor Biology, University Medical Center Hamburg-Eppendorf, Hamburg, Germany; ^5^ Department of Neurosurgery, Klinikum Grosshadern, Ludwigs-Maximilians-University, Munich, Germany; ^6^ Institute of Neuropathology, University Medical Center Hamburg-Eppendorf, Hamburg, Germany; ^7^ Department of Neuropathology, Institute of Pathology, Heidelberg, Germany; ^8^ Clinical Cooperation Unit Neuropathology, German Cancer Consortium (DKTK), German Cancer Research Center (DKFZ), Heidelberg, Germany; ^9^ Institute of Medical Biometry and Informatics, University of Heidelberg, Heidelberg, Germany; ^10^ Research Group Molecular Genetics of Breast Cancer, German Cancer Research Centre (DKFZ), Heidelberg, Germany

**Keywords:** glioblastoma, serum, antibodies, long-term survival, TNC, non-invasive biomarker

## Abstract

Liquid biopsies come of age offering unexploited potential to monitor and react to tumor evolution. We developed a cost-effective assay to non-invasively determine the immune status of glioblastoma (GBM) patients. Employing newly developed printed peptide microarrays we assessed the B-cell response against tumor-associated antigens (TAAs) in 214 patients. Firstly, sera of long-term (36+ months, LTS, n=10) and short-term (6-10 months, STS, n=14) surviving patients were screened for prognostic antibodies against 1745 13-mer peptides covering known TAAs (TNC, EGFR, GLEA2, PHF3, FABP5, MAGEA3). Next, survival associations were investigated in two retrospective independent multicenter validation sets (n=61, n=129, all IDH1-wildtype). Reliability of measurements was tested using a second array technology (spotted arrays). LTS/STS screening analyses identified 106 differential antibody responses. Evaluating the Top30 peptides in validation set 1 revealed three prognostic peptides. Prediction of TNC peptide VCEDGFTGPDCAE was confirmed in a second set (p=0.043, HR=0.66 [0.44-0.99]) and was unrelated to TNC protein expression. Median signals of printed arrays correlated with pre-synthesized spotted microarrays (p<0.0002, R=0.33). Multiple survival analysis revealed independence of age, gender, KPI and MGMT status. We present a novel peptide microarray immune assay that identified increased anti-TNC VCEDGFTGPDCAE serum antibody titer as a promising non-invasive biomarker for prolonged survival.

## INTRODUCTION

Glioblastoma (GBM), the most common primary brain tumor, ranks among the deadliest human cancers [1]. Despite current standard therapy consisting of a maximal safe resection followed by radiotherapy and chemotherapy with temozolomide, the prognosis remains dismal with a median overall survival (OS) of about 15 months [2, 3]. Seeking to identify new therapeutic targets, large-scale consortia have been comprehensively characterizing the genetic and transcriptional landscape of GBMs [4, 5]. Although this data let to considerable progress in the understanding of gliomagenesis, the observed extent of heterogeneity between glioblastomas was a setback in the endeavor to find novel therapeutic targets for the majority of patients. However, new hope was risen by recent pioneering studies indicating that the immune system in glioblastomas, contrary to popular opinion for decades, actively contributes to tumor emergence, editing and progression [6, 7]. In parallel, peptidomic and proteomic analyses have been identifying an increasing number of tumor-associated antigens (TAAs) [8-10]. Immune responses against TAAs can primarily arise by (i) a reexpression of genes of embryonic development (oncofetal antigens), (ii) a marked overexpression upon gliomagenesis or (iii) a changed amino acid sequence (neoantigens) [11]. Due to these characteristics private to the tumor antigen repertoire, TAAs are known to be targets of both the humoral and cell-mediated immune response. As a consequence, no high-grade primary brain tumor is considered to evolve without harboring multiple immunosuppressive mechanisms. However, given the profound inter-tumoral heterogeneity observed in glioblastomas, it is likely that the anti-tumor response of immune systems significantly differs between patients.

Developing meaningful immune assays to determine the immune status of a patient is very appealing as they not only promise to be powerful prognostic biomarkers but if correctly applied also enable patient stratification for the increasing number of immunotherapeutical trials in brain tumor patients (reviewed in [12]). The ideal immune assay would be non-invasive, enabling a monitoring of the immune status of a patient over time. As anti-tumor T-cell responses are more difficult to quantify in a timely and high-throughput manner due to e.g. the need of higher blood volumes, investigating anti-tumor B-cell might prove a promising alternative. Although little is known about the B-cell response towards TAAs in GBM, anti-tumor antibodies could be observed in GBMs [13]. If antibodies against TAA in GBM robustly correlate to tumor burden or predict the course of the disease remains elusive.

An astute way to non-invasively monitor antibody responses are peptide or protein microarrays [14, 15]. Due to their miniature format they allow for the multiplex analysis of several thousands of peptides at the same time while requiring a minimal sample volume [16]. Here, recently developed laser-printed peptide arrays uniquely offer a fast and cost-effective way for the combinatorial synthesis of peptide arrays [17]. A prime challenge remains the choice of antigens, as it is up to date impossible to cover the whole linear proteome using peptide microarrays.

In glioblastomas, the growing list of candidate TAAs include the epidermal growth factor receptor (EGFR) [18], tenascin-C (TNC) [19], fatty acid binding protein 5 (FABP5) [20], melanoma-associated antigen 3 (MAGEA3) [21], glioma-expressed antigen 2 (GLEA2) [22, 23], and PHD finger protein 3 (PHF3) [23, 24]. Among these, especially the extracellular matrix protein TNC has been known as glioma-associated antigen for decades and its contribution to gliomagenesis has been extensively studied [25]. Physiologically it is expressed in embryogenesis and wound healing and is almost absent in normal brain underscoring its relevance as one of the most important TAAs in GBM [19, 26, 27]. In GBM it is highly expressed and promotes tumor cell invasion *in vitro* and *in vivo* and immunosuppression by inhibiting the polarization and transmigration of T-cells [28-30]. In addition, its antigenic potential has been exploited as part of a peptide vaccine [9], which could be shown to be safely applied and to elicited specific T-cell responses in the majority of GBM patients (reviewed in [31]).

Regarding EGFR, frequent overexpression and gene amplification have been shown as a major characteristics of primary GBM [18]. Furthermore, in a very recent publication, the implication of wildtype EGFR and EGFR deletion variants for important hallmarks of GBM biology such as invasion and angiogenesis has been elegantly demonstrated *in vivo* [32]. For the cancer testis antigen MAGEA3, both a GBM-specific overexpression as well as antibody responses in gastric cancer have been described [21]. Finally, in serological analyses by the SEREX (Serological analysis of expression cDNA libraries) technology GLEA2 and PHF3 were found to frequently elicit immune responses in sera of GBM patients [22-24].

Applying innovative printed peptide microarrays we successfully developed the first TAA-based non-invasive immune assay for glioblastoma patients. An increased titer of antibodies against a previously undescribed epitope within the TNC molecule was identified to predict prolonged survival independent from known prognostic clinicopathological parameters. To our knowledge, our study is also the first to use large-scale multi-center IDH1-wildtype glioblastoma study sets for which all necessary clinical data were available to enable meaningful multiple survival analysis. The cost-effective miniature format and the extremely low sample volume further underline the great promise of this analytical workflow to monitor the immune response of patients within clinical studies.

## RESULTS

### Differential serum autoantibodies in LTS and STS GBM patients

**Figure 1:**
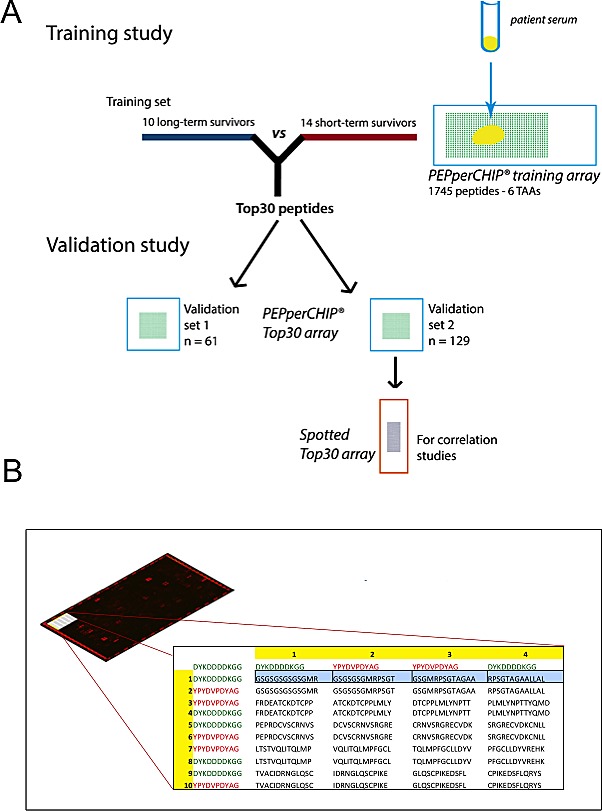
(**A**) Graphical abstract of study design. Firstly, a training study was conducted to identify candidate prognostic autoantibodies. To this end, sera of 10 long-term surviving and 14 short-term surviving patients were incubated on peptide microarrays covering the linear amino acid sequence of 6 tumor-associated antigens (1745 peptides). The Top30 peptides showing the highest differential antibody response were then validated in two independent multicenter study cohorts of together 190 samples. Reliability of antibody measurements were validated by retesting all samples of validation set 2 (*n* = 129) with peptide microarrays generated by a different technology (pre-synthesized spotted arrays). (**B**) Schematic design of the customized PEPperCHIP^®^ screening microarray. In the top left of the Figure, a representative array scan is depicted. A red fluorescent labeled secondary antibody binding to the human heavy chain visualized patient antibodies specifically bound to spotted peptides on the array. The red spots on the border of the array denote control spots. The table illustrates an extract of the array design in the upper left corner of the array. Overlapping 13 amino acids peptides (overlap of 9 amino acids) were printed as duplicates together with 244 control peptides (HA and FLAG epitopes; red and green font) to the array.

**Figure 2:**
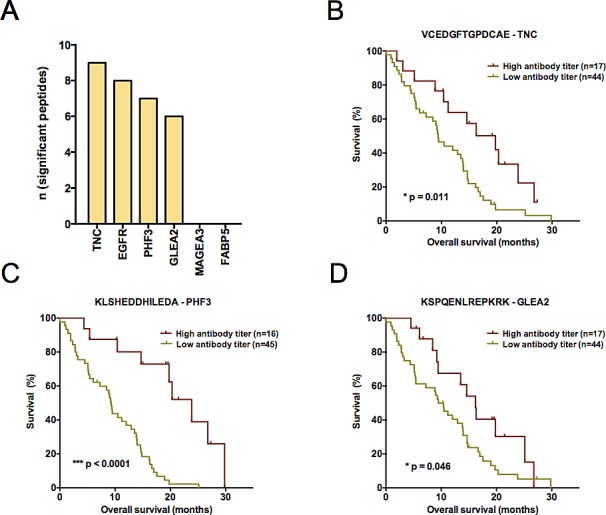
(**A**) Composition of the Top30 peptides identified by comparative analysis of long- and short-term surviving patients. Barplots depict the absolute number of peptides per antigen. For FASTA sequences of antigens see supplemental material and methods. (**B-D**) Kaplan-Meier plots visualizing antibody titers with a significant predictive performance in the first validation study set (*n* = 61). Antibody responses against (**B**) VCEDGFTGPDCAE – TNC (**C**) KLSHEDDHILEDA – PHF3 and (**D**) KSPQENLREPKRK – GLEA2 could be identified to significantly predict patient survival (overall survival). A high antibody titer denotes a signal intensity belonging to the 1st quartile of ranked signal intensities on the Top30 printed peptide array. Log-rank *p*-value is given in each plot.

In search for novel non-invasive GBM biomarkers we applied the PEPperPRINT^®^ technology and designed customized printed peptide arrays covering the complete linear amino acid sequence of six known tumor-associated antigens (EGFR [18], TNC [19], GLEA2 [22, 23], MAGEA3 [21], PHF3 [23, 24], FABP5 [20]; Figure 1B, data supplement). To identify prognostic circulating serum autoantibodies, we compared their titers in patients with large survival differences. Our screening set contained 10 long-term (LTS) and 14 short-term surviving (STS) patients (Figure 1A). We observed polyclonal antibody responses against all TAAs printed to the screening array. Statistical analyses revealed 106 differential antibody responses (multiplicity unadjusted *p* < 0.05). For 57 of these peptides we observed an increased antibody titer in LTS patients, and for 49 peptides in STS patients. Next, we compared median signal intensities of all antibody titers targeting a respective antigen. Here, median signal intensity for MAGEA3 was significantly higher in LTS, whereas median signal intensity did not differ for the other TAAs (*p* = 0.0025; Suppl. Figure 2). However, a detailed analysis of all 75 MAGEA3 peptides on the screening array revealed only a week inter-peptide correlation (Suppl. Figure 3) suggesting a poor classification power for MAGEA3. Indeed, the Top30 differential antibody responses of all TAAs (Suppl. Table 2) showed in a principal component analysis (PCA) superior grouping of LTS and STS patients compared to MAGEA3 peptides (Suppl. Figure 4). Therefore, we selected the Top30 peptides with lowest probability values (Suppl. Table 2) for testing in 2 independent validation sets. Noteworthy, they corresponded to only 4 of the 6 tested antigens (TNC, *n* = 9; EGFR, *n* = 8; PHF3, *n* = 7; GLEA2, *n* = 6; Figure 2A). Among the Top30 peptides, 13 antibody responses had a higher median titer in LTS and 17 in STS patients.

### Identification of prognostic serum antibodies in independent validation sets

Again employing the PEPperPRINT^®^ technology, we created a customized Top30 candidate peptide array (Figure 1A). Signal intensities of Top30 peptides were ranked to perform inter-array normalization. An antibody titer was considered “increased”, if it ranked in the 1^st^ quartile of all signal intensities measured on the patient's Top30 array. If antibody titers against all 30 peptides for a patient were low (<25% of median signals over all patients), response intensities for this patient were ranked on the 30th place. To investigate the prognostic value for GBM patients, we assessed antibody titers against Top30 peptides in a first independent multi-institutional 61-patient validation set, which resembled normal overall survival (OS) characteristics. Here, OS was not associated with the clinicopathological parameters age, gender, KPI, MGMT promoter methylation status and chemotherapy (Table 2). As expected, patients receiving radiotherapy (46/61 patients) presented with a significantly improved OS. Differing number of patients receiving adjuvant radiotherapy across the study samples might explain the observed survival difference between centers. Altogether, high antibody titers against 3 peptides were found to be significant prognosticators of patient survival (Figure 2B-2D). Peptides corresponded to TNC (VCEDGFTGPDCAE; 17 patients with an increased response, *p* = 0.011, HR = 0.43), PHF3 (KLSHEDDHILEDA; 16 patients with an increased response, *p* = 3.67E-05, HR = 0.17) and GLEA2 (KSPQENLREPKRK; *p* = 0.05, HR = 0.52). For all three targeted peptides, an increased titer was prognostic for a better patient survival. In multiple regression survival analysis, an increased antibody titer against KLSHEDDHILEDA remained statistically significant (Table 3).

**Table 2:** Univariate analysis of confounders and candidate peptides in validation sets Results of Cox proportional hazard analysis are summarized. *P*-value were calculated employing log-rank test (*p < 0.05, **p < 0.01, ***p < 0.001). To enable interpretation of correlation between antibody titer and patient survival, the median overall survival (OS) for patients with a high and low titer is listed. For all peptides, a high antibody titer was associated with a prolonged patient survival.

	HR	95% CI	*p*-value	median OS high titer (months)	median OS low titer (months)
**Validation set 1**					
age	0.71	0.41-1.24	0.229		
gender	0.65	0.37-1.15	0.138		
KPI	0.99	0.98-1.02	0.664		
MGMT	0.59	0.22-1.60	0.291		
radiotherapy	0.43	0.23-0.80	6.68E-03**		
chemotherapy	1.28	0.45-3.61	0.643		
study center - Municht†	0.33	0.16-0.68	2.87E-03**		
study center - Hamburg†	2.18	1.07-4.43	0.031*		
VCEDGFTGPDCAE (TNC)	0.43	0.22-0.84	0.011*	19.76	9.44
KLSHEDDHI LEDA (PHF3)	0.17	0.07-0.40	3.67E-05***	23.84	9.30
KSPQENLREPKRK (GLEA2)	0.52	0.27-0.99	0.046*	16.21	9.96
**Validation set 2**					
age	0.59	0.40-0.85	5.42E-03**		
gender	1.27	0.87-1.86	0.215		
KPI	0.99	0.98-1.01	0.614		
MGMT	0.67	0.43-1.04	0.073		
study center - Munich †	0.57	0.30-1.07	0.08		
study center - Hamburg †	0.72	0.39-1.33	0.29		
VCEDGFTGPDCAE (TNC)	0.66	0.44-0.99	0.043*	18.44	15.00
KLICSEKGKVSEK (GLEA2)	0.65	0.42-0.99	0.048*	20.12	14.96	

Patients were dichotomized regarding age into younger or older than median age in the respective validation set. Abbreviations: KPI = preoperative Karnofsky Performance Index; HR = hazard ratio; CI = confidence interval; OS = overall survival; MGMT = promotor methylation of O-6-methylguanine-DNA methyltransferase.

† Cox proportional hazard analysis for study centers was performed in comparison to Heidelberg study samples.

**Table 3:** Multiple survival analysis of candidate antibody responses in validation sets For multiple survival analysis, all clinicopathological confounders significant in the univariate analysis were included in the multivariate model. Results of Cox proportional hazard analysis are summarized (*p < 0.05, **p < 0.01, ***p < 0.001).

	HR	95% CI	*p*-value
**Validation set 1**			
radiotherapy	0.64	0.32-1.26	0.194
study center - Municht†	0.54	0.22-1.35	0.187
study center - Hamburg, †	2.00	0.95-4.21	0.068
VCEDGFTGPDCAE (TNC)	1.35	0.56-3.22	0.504
KLSHEDDH1LEDA (PHF3)	0.16	0.05-0.50	1.54E-03**
KSPQENLREPKRK (GLEA2)	0.66	0.29-1.49	0.315
**Validation set 2**			
Age	1.03	1.01-1.04	5.88E-03**
VCEDGFTGPDCAE (TNC)	0.68	0.45-1.02	0.0487*

Patients were dichotomized regarding age into younger or older than median age in the respective validation set. Abbreviations: KPI = Karnofsky Performance Index; HR = hazard ratio; CI = confidence interval.

† Cox proportional hazard analysis for study centers was performed in comparison to Heidelberg study samples.

**Figure 3:**
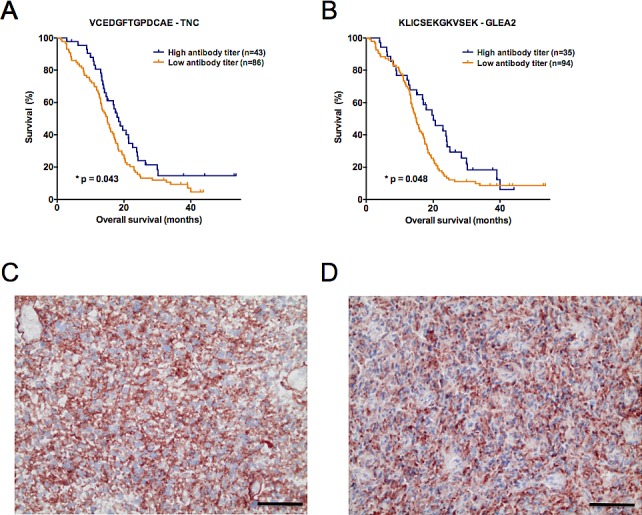
(**A, B**) Kaplan-Meier plots illustrating the antibody titers with a significant predictive performance in the second validation study set (n = 129). Autoantibodies against (**A**) VCEDGFTGPDCAE – TNC and (**B**) KLICSEKGKVSEK – GLEA2 significantly predicted patient survival (overall survival). As for validation set 1, a high antibody titer denotes a signal intensity belonging to the 1st quartile of ranked signal intensities on the Top30 printed peptide array. Log-rank *p*-value is given in each plot. (**C**, **D**) Protein expression of tenascin-C (red color) analyzed by immunohistochemistry on cryosections of long- and short-term surviving GBM patients. Representative stainings in (**C**) LTS and (**D**) STS patients are shown. Black scale bar denotes 50 μm.

To challenge the prognostic performance of our candidate peptides we analyzed an additional 129 patients with the same Top30 peptide microarray. Validation set 2 was independent from validation set 1 and again combined samples from all study sites. Importantly, all samples were molecularly tested for IDH1 mutations and only IDH1-wildtype patient tumors included. The major difference to validation set 1 was that all patients received a uniform adjuvant treatment according to the EORTC/NCIC-protocol (radio- and chemotherapy). After having observed promising results in the first validation set, we deliberately choose a uniformly treated second study set to enable an even more meaningful multiple regression analysis. This is achieved by reducing the number of confounders in the multivariate model. Confirming the results of validation set 1, antibody responses against TNC peptide VCEDGFTGPDCAE were again prognostic for a better OS in validation set 2 (*p* = 0.043, HR = 0.66 [0.44-0.99]; Figure 3A). Furthermore, another GLEA2 peptide (KLICSEKGKVSEK) was identified to be prognostic in validation set 2 (Table 2; Figure 3B). In contrast to the first validation set, age was a significant confounder of patient survival (Table 2). In subsequent multivariate analysis, however, only TNC peptide VCEDGFTGPDCAE remained significant when adjusted for patient age (Table 3).

### Correlation of printed peptide array signals with independent spotted peptide arrays

The feasibility of implementing novel biomarkers into clinical practice depends on their reliable detection across different technology platforms. To investigate, if measured antibody titers are unrestricted to printed peptide microarrays, we compared them to measurements from peptide microarrays of different architecture. Using validation set 2, we generated a second set of microarrays by spotting pre-synthesized and purified peptides on glass slides. Median signal intensity of Top30 PEPperCHIP^®^ microarrays significantly correlated with median signal intensity of the Top30 spotted microarrays (*p* < 0.0002, R = 0.29; Suppl. Figure 5A). Moreover, intensities of the TNC peptide VCEDGFTGPDCAE were significantly correlated between the two microarray platforms (*p* < 0.0008, R = 0.29; Suppl. Figure 5B).

### TNC protein expression in LTS and STS patients

To explore if differing titers of TNC autoantibodies in LTS and STS are due to differences of TNC protein expression in the original tumor tissue, we performed immunohistochemistry in our initial screening set. Comparing the stainings of 13 STS and 10 LTS patients revealed a marked TNC protein expression in all cases and no difference between LTS and STS patients (Figure 3C-3D). TNC showed a strong expression in the tumor center, whereas it was very weak or absent in the tumor margin (data not shown).

## DISCUSSION

Applying innovative printed peptide arrays, we developed a non-invasive 30-peptide array to investigate the anti-tumor B-cell response in GBM patients and identified prognostic serum antibody responses against the TNC peptide VCEDGFTGPDCAE. The prognostic capability of this antibody response could not only be demonstrated in three different independent study samples in a multicenter setting but was also identified to be independent from known prognostic confounders and the extent of TNC protein expression in the tumor tissue. Moreover, the robustness of our approach could be confirmed by an independent technological platform.

It is an interesting finding that especially the TNC peptide VCEDGFTGPDCAE has a predictive value in both validation study samples. This is not surprising as it was already the most differentially targeted peptide in our initial discovery cohort of short- and long-term surviving GBM patients (see Suppl. Table 2). The role of our novel antibody target VCEDGFTGPDCAE is corroborated by the fact that the TNC protein is exclusively upregulated under pathological conditions. Tumor-specific overexpression of TNC might also be responsible for the highest numbers of peptides targeted by antibody responses among the TAAs analyzed in our study (Figure 2A). An explanation why only the VCEDGFTGPDCAE peptide could be confirmed to be survival-associated in all three data sets could be its location within the TNC molecule. This peptide is part of the EGF-like domain (UniProt entry P24821, positions 517-528) and EGF-like repeats of TNC were shown to support cell proliferation by EGFR autophosphorylation in an EGFR-dependent manner [33]. Noteworthy, unlike literature reports describing that TNC expression in gliomas increases with the WHO grade [19] and that few GBM patients with a lower protein expression presented with an increased survival (28 months) [34], we did not observe a differential expression in our screening set consisting of LTS and STS GBM patients. In contrast, we found a strong and almost homogeneous expression in all samples analyzed. Since most of the data on TNC expression did not assess the prognostically relevant isocitrate dehydrogenase 1 (IDH1) mutation status, an enrichment of IDH1-mutant GBM with a better outcome in these previous analyses cannot be excluded. Therefore we assume that the observed survival-associated antibody responses against the VCEDGFTGPDCAE peptide are more likely caused by biological differences of the patients' immune system rather than by expression differences of the TNC protein in IDH1-wildtype GBM. It will be of particular interest to investigate the titer of anti-TNC peptide VCEDGFTGPDCAE before and after immunotherapies, as first strategies targeting TNC by immunotherapy are being established within clinical studies [35].

Serum biomarkers have gained broad attention, since they could non-invasively support preoperative treatment decisions and would facilitate monitoring the course of the disease. However, the majority of studies in GBM patients only discriminate between tumor and healthy controls or included low patient numbers. Most importantly, so far none of these studies confirmed their data in independent study sets. For instance, in newly diagnosed GBMs significantly elevated serum levels of the glycan-binding protein Galectin-1 and glial fibrillary acidic protein (GFAP) [36] have been identified by ELISA and upon future validation could serve as diagnostic tools. Regarding patient survival, in a small study set of 30 GBM patients, Osteopontin serum levels [37] and more recently, autoantibody responses in 40 GBM patients against URGCP were shown to be prognostic [38]. However, the value of both results might be limited since only univariate analyses were conducted. Only few studies performed multivariate analyses. For example, an exceptionally large retrospective study consisting of 549 GBM patients suggested pre-operative serum albumin levels to be an independent predictor of survival (*p* < 0.005), yet with little impact (HR 0.97) [39]. Also, low serum concentrations of the glycoprotein α_2_-HS have been shown to be associated with an improved survival in 91 GBM patients independent of age and KPI [40]. Finally, a small study (*n* = 36) discovered TIMP-1 serum levels to predict survival independent of age, KPI, and treatment [41]. Noteworthy, none of the studies included nowadays as essentially acknowledged important prognostic variables such as IDH1 mutation or MGMT promoter hypermethylation status in their multivariate models. It is also questionable how the lack of independent validation sets impacts on these findings, because our analysis clearly demonstrates the importance of i) a multivariate analysis including all known prognostic confounders and ii) the use of multiple independent study sets. Along this line of reasoning, we found that not all prognostic peptides from the univariate analysis remained significant in subsequent multivariate analysis and that only a subset of peptides can be validated in further independent study samples.

Moreover, our study is exceptional as both of our validation sets exclusively consisted of IDH1-wildtype GBM patients. In addition, our second validation set contained a homogeneous patient group reflecting the current standard of care. To date, a considerable fraction of GBM long-term survivors was identified to harbor IDH1 mutations [42, 43]. Comprehensive work on the biology of IDH1-mutant GBM has led to the conclusion that even without detectable precursor lesions they seem to belong to the group of secondary GBM [44]. That they differ markedly from IDH1-wildtype primary GBM, is further reflected by the observed survival differences in favor of IDH1-mutant GBM [45-48]. Hence, it is likely that biomarker studies in GBMs over the last years have been heavily biased towards IDH1-mutant tumors and thus primarily focused on differences between IDH1-wildtype and IDH1-mutant GBM. Therefore, our study constitutes an important contribution to our understanding about biomarkers associated with an improved survival in IDH1-wildtype GBMs. Nevertheless, we cannot entirely exclude to have lost the two other prognostic peptides of validation set 1 (KLSHEDDHILEDA (PHF3) & KSPQENLREPKRK (GLEA2) due to the fact that patients in validation set 2 were treated more homogeneously. How this impacts on the prognostic value of antibody responses should be addressed in future investigations.

In summary, we successfully developed a non-invasive 30-peptide array to investigate the anti-tumor B-cell response in sera of GBM patients. Using this assay, we discovered a novel epitope within the TNC molecule that is frequently recognized by circulating antibodies. Due to its survival association higher anti-VCEDGFTGPDCAE antibody titers could serve as an independent non-invasive biomarker. Moreover, our validation sets of together 190 IDH1-wildtype GBM patients represent one of the most robust study samples available, enabling meaningful multivariate analyses which are unbiased by the dominant phenotype of IDH1-mutant GBM. Finally, the cost-effective miniature format of only 30 peptides per microarray and the extremely low sample volume of only 1μl uniquely offer future inclusion of this type of serum analysis in clinical trials.

## MATERIALs AND METHODS

### Study samples

*LTS/STS screening set.* Our discovery cohort consisted of 10 long-term (>36 months) and 14 short-term (6-10 months) surviving GBM patients, who underwent bulk tumor resection at Dpt. Neurosurgery, Heidelberg University Hospital, Germany and died from tumor-related death. The majority of patients received post-operative radio- and chemotherapy, followed by adjuvant temozolomide according to EORTC/NCIC protocol. Clinicopathological characteristics are summarized in Suppl. Table 1. Except for survival, there was no significant difference regarding known prognostic confounders (age, gender, MGMT promoter methylation, Karnofsky performance index (KPI)) between LTS and STS patients.

*Validation sets.* Validation set 1 was composed of 61 and set 2 of 129 GBM patients receiving bulk tumor resection at three different German centers (Neurosurgery Department Heidelberg, Hamburg, and Munich). Clinicopathological characteristics are summarized in Table 1. While in validation set 1 all patients with available clinical data were included independent of their adjuvant treatment for set 2 only patients were selected who received the current standard of care consisting of a combined radio- chemotherapy. IDH1 mutation was routinely excluded by either immunohistochemistry or sequencing.

**Table 1:** Clinicopathological characteristics of validation sets All sera were obtained preoperatively from therapy-naïve glioblastoma patients. Board-certified neuropathologists verified diagnosis of all patients. Validation set 2 included only patients that received adjuvant radio-chemotherapy. Importantly, all patients did not harbor a R132H mutation in the isocitrate dehydrogenase 1 (IDH1) gene.

	Validation set 1 (n=61)		Validation set 2 (n=129)	
Characteristic	No.	%	No.	%
Age, years				
Median	69		60	
Range	34-84		17-77	
Gender				
Male	39	64	77	60
Female	22	36	52	40
Treatment				
Surgery	61	100	129	100
No chemo- or radiotherapy	15	25	0	0
Chemotherapy	0	0	0	0
Radiotherapy	41	67	0	0
Radio-chemotherapy	5	8	129	100
Overall survival				
Median	10		15	
Range	0-30		1-54	
MGMT				
Hypermethylated	10	16	41	32
Unmethylated	15	25	49	38
Unknown	36	59	39	30
IDH I				
wildtype	61	100	129	100
unknown	0	0	0	0
KPI				
100%-80%	46	75	106	82
70%-50%	14	23	22	17
40%-20%	1	2	0	0
Unknown	0	0	1	1
Study Center				
Heidelberg	32	52	101	78
Hamburg	12	20	14	11
Munich	17	28	14	11

Abbreviations: KPI = preoperative Karnofsky Performance Index; No. = number of cases; IDH1 = mutation in isocitrate dehydrogenase 1 (R132H). MGMT = promotor methylation of O-6-methylguanine-DNA methyltransferase.

### Ethical approval

Study was approved by relevant ethical committees in all study centers. Written informed consent was obtained from all patients.

### Serum preparation

Blood samples were acquired pre-operatively. Heidelberg and Munich samples were collected in serum and Hamburg samples in citrate tubes. Serum or plasma were separated by centrifugation (10 minutes at 5.000 rpm), aliquoted and stored at −20°C or below.

### Peptide microarray generation

Amino acid sequences of human proteins EGFR, FABP5, GLEA2, MAGEA3, PHF3 and Tenascin-C (for FASTA sequences see Suppl. Material) were translated into 13-mer peptides with a peptide-peptide overlap of 9 amino acids. On-chip combinatorial peptide synthesis was done by laser printing technology on glass slides coated with a PEGMA/PMMA graft copolymer [17] and functionalized with a dipeptidic ßAla-ßAla-linker. In brief, one layer of amino acid toner particles with 10% w/w Fmoc-amino acid pentafluorophenyl esters was printed with high resolution onto the functionalized glass slides followed by melting at 90°C to release the activated amino acids and to initiate coupling. After standard capping, washing and deprotection steps according to Merrifield, the next layer of amino acid toner particles was printed on top of the first amino acid layer with high spatial resolution. The process was repeated until the intended peptide length was reached. The resulting PEPperCHIP^®^ training peptide microarrays (3′’ × 1′’, 75.4 mm × 25.0 mm × 1 mm) contained two identical array copies with 1745 different peptides printed in duplicate (3490 peptide spots) that were framed by 122 Flag (DYKDDDDKGG) and 122 HA (YPYDVPDYAG) control peptides (Figure 1B).

For the validation study, 30 top binding peptides of the first screening round were synthesized as 13-mer peptides in duplicate [17] on PEPperCHIP^®^ peptide microarrays with 16 identical array copies.

Technical validation was done with peptide arrays based on pre-synthesized and purified peptides. Synthesis of these peptides was performed by an AutoSpot robot (INTAVIS Bioanalytical Instruments AG, Cologne, Germany) according to standard solid phase Fmoc-chemistry. Peptides were purified according to standard procedures and quality control was carried out by RP-HPLC (1260 Infinity System, Agilent, Waldbronn, Germany) and MALDI-TOF mass spectrometry (Reflex II, Bruker-Daltonik, Bremen, Germany). Spotting was performed on glass slides with an SDDC-2 DNA Micro-Arrayer from Engineering Services Inc. (Toronto, Canada). Each slide contained 16 identical array copies with the 30 top binding peptides in duplicates.

### Immune staining of microarrays

PEPperCHIP^®^ peptide microarrays were placed in suited PEPperCHIP^®^ incubation trays (PEPperPRINT GmbH, Heidelberg, Germany) and blocked for 1 h at room temperature at 200 RPM orbital shaking with blocking buffer MB-070 (Rockland, Gilbertsville, PA, USA). Next, sera were diluted 1:100 in 1 × PBS buffer with 0.05% Tween 20 pH 7.4 (PBST) and 10% MB-070 followed by incubation for 16 h at 4 °C and 500 RPM orbital shaking. For the training array (1745 peptides) 10 μl of serum was used and for the validation array (30 peptides) 1 μl. Peptide microarrays were washed 3 × 1 min with PBST followed by an incubation with a 1:5000 dilution of the secondary antibody (anti-human IgG (H+L) DyLight 680, Rockland, Gilbertsville, PA, cat# 609-144-123) for 30 min at room temperature and 200 RPM orbital shaking. The peptide microarrays were washed 3 × 1 min with PBST and rinsed with deionized water. After drying in a stream of air, images were recorded using an Odyssey Imaging System (LI-COR, Lincoln, NE, USA) at a wavelength of 700 nm, a resolution of 21 μm and a scanning sensitivity of 7.

For staining of HA and Flag control peptides, arrays were pre-swollen for 10 min in PBST at room temperature followed by incubation with a mixture of 1:1000 dilutions of monoclonal anti-HA (12CA5)-LL-DyLight680 and monoclonal anti-FLAG(M2)-LL-DyLight800 (PEPperPRINT GmbH, Heidelberg, Germany) in 10% MB-070/PBST for 1 h at room temperature and 200 RPM orbital shaking. After washing for 3 × 1 min with PBST and rinsing with deionized water, slides were dried and images were recorded at 700 and 800 nm with a resolution of 21 μm and a scanning sensitivity of 7 for each channel.

Immune staining of spotted peptide arrays was done as above except for the use of 50% NAP-blocker (G-Biosciences, St. Louis, MO, USA) in PBST + 1% Triton X100 pH 7.4 instead of MB-070 for blocking and antibody incubation. Image analysis and quantification of array data were done with PepSlide^®^ Analyzer (Sicasys Software GmbH, Heidelberg, Germany). Suppl. Figure 1 illustrates representative stainings of the training array.

### Immunohistochemistry

Immunohistochemical staining was performed as described [19]. Mouse monoclonal TNC antibody (clone TN2, DAKO) was diluted with Antibody Diluent (DAKO) to a concentration of 13 μg/ml and incubated for 1hr at room temperature on 5 μm cryosections. Specificity of primary antibody was controlled using an isotope control antibody (IgG1, Acris) at the same concentration.

### Statistical analysis

Raw-intensity data of screening 1745 PEPperCHIP^®^ microarray was first inter-array normalized using quantile normalization, followed by intra-array normalization using median-centering. Lastly, intensity data was log2 transformed. Intensity signals for peptides of the Top30 validation array were ranked for comparative analysis. Differential antibody responses in LTS and STS patients were assessed using Student's t-tests. For survival analyses, patients were followed-up from operation to death. Survival association with individual peptide and multipeptide combinations was analyzed using univariate log-rank tests and multiple Cox regression models, including all clinicopathological parameters statistically significant in univariate analyses. Statistical analyses were conducted in *R (www.r-project.org).* Survival analyses were performed employing the survival package.

## SUPPLEMENTARY MATERIALS TABLES AND FIGURES



## Abbreviations

GBM: glioblastoma
TAA: tumor-associated antigen
LTS: long-term survivor
STS: short-term survivor
TNC: tenascin-C
EGFR: epidermal growth factor receptor
GLEA2: glioma-expressed antigen 2
PHF3: PHD finger protein 3
FABP5: fatty acid binding protein 5
MAGEA3: melanoma-associated antigen 3
KPI: Karnovsky performance index
MGMT: O-6-methylguanine-DNA methyltransferase
OS: overall survival
IDH1: isocitrate dehydrogenase 1
