# Supplementary material for: Printed peptide arrays identify prognostic TNC serumantibodies in glioblastoma patients
Source: Oncotarget. 2015 Apr 12;6(15):13579–90. doi: 10.18632/oncotarget.3791 (PMC4537035; doi:10.18632/oncotarget.3791)
Supplement: Supplementary file 1 [file oncotarget-06-13579-s001.pdf]

**Printed peptide arrays identify prognostic TNC serumantibodies in glioblastoma patients**

# Supplementary Material

**Supplemental Table 1:** Clinicopathological characteristics of screening set consisting of 10 long-term and 14 short-term surviving GBM patients. All sera were obtained preoperatively from therapy-naïve patients. Board-certified neuropathologists verified diagnosis of all tumor specimens. The majority of patients received adjuvant radio-chemotherapy. *p<0.05, **p<0.01, ***p<0.001.

| Characteristic | Long-term survivor (LTS) | | Short-term survivor (STS) | | |
|---|---|---|---|---|---|
| | No. | % | No. | % | p-value |
| Case number | 10 | | 14 | | |
| Histological diagnosis | | | | | |
| Glioblastoma | 10 | 100 | 14 | 100 | |
| Age (months) | | | | | |
| Median | 59 | | 63 | | |
| Range | 36-67 | | 33-81 | | 0.371 |
| Gender | | | | | |
| Male | 5 | 50 | 10 | 71 | |
| Female | 5 | 50 | 4 | 29 | 0.403 |
| Treatment | | | | | |
| Surgery | 10 | 100 | 14 | 100 | |
| No radio- or chemotherapy | 0 | 0 | 1 | 7 | |
| Chemotherapy only | 0 | 0 | 2 | 14.3 | |
| Radiotherapy only | 1 | 10 | 2 | 14.3 | |
| Radio-chemotherapy | 9 | 90 | 9 | 64.3 | |
| Overall survival (months) | | | | | |
| Median | 58 | | 8 | | |
| Range | 42-185 | | 6-10 | | 1.88E-05*** |
| MGMT | | | | | |
| Hypermethylated | 7 | 70 | 8 | 57 | |
| Unmethylated | 2 | 20 | 4 | 27 | |
| Unknown | 1 | 10 | 2 | 16 | 0.659 |
| IDH1 | | | | | |
| wild-type | 9 | 90 | 14 | 100 | |
| mutant (132H) | 1 | 10 | 0 | 0 | 0.417 |
| KPI | | | | | |
| 100%-90% | 6 | 60 | 12 | 86 | |
| 80%-70% | 3 | 30 | 2 | 14 | |
| Unknown | 1 | 10 | 0 | 0 | 0.343 |
| Study Center | | | | | |
| Heidelberg | 10 | 100 | 14 | 100 | |

Abbreviations: KPI = preoperative Karnofsky Performance Index; LTS = long-term survivor; STS = short-term survivor; IDH1 = mutation in isocitrate dehydrogenase 1 (R132H); MGMT = promotor methylation of O-6-methylguanine-DNA methyltransferase. Statistical tests: Student's t-test (age, overall survival), fisher's exact test (gender, MGMT, IDH1, KPI), No. = number of cases

**Supplemental Table 2** : Differential antibody abundance for the Top30 peptides in LTS and STS patients. Peptides are assigned to their corresponding antigen and ordered according to their p-value. A positive log2 fold change (log2 FC) and difference in signal intensity equals a higher titer in LTS patients.

| Peptide | Antigen | P-Value | Intensity difference | log2 FC |
|---|---|---|---|---|
| VCEDGFTGPDCAE | TNC | 0.000633244 | 1570.269 | 0.8745 |
| KLICSEKGKVSEK | GLEA2 | 0.00065385 | -611.7965 | -0.578 |
| KETVKLSHEDDHI | PHF3 | 0.001009832 | 692.643 | 0.5615 |
| VSGNTVEYALTDL | TNC | 0.001521112 | 1303.234 | 0.7635 |
| QAHSVLKKTLQDQ | PHF3 | 0.003270692 | 566.2445 | 0.4935 |
| TGFLLIQAWPENR | EGFR | 0.003687734 | -713.4405 | -0.6515 |
| KKLFGTSGQKTKI | EGFR | 0.004568125 | -1066.191 | -0.9985 |
| KVVDCLKFFRKAK | GLEA2 | 0.004801176 | -1280.414 | -0.8375 |
| EGKYSFGATCVKK | EGFR | 0.00611762 | -1535.3945 | -1.0485 |
| KLSHEDDHILEDA | PHF3 | 0.006157174 | 1806.9675 | 0.8915 |
| KDKEKNKEKKFKE | GLEA2 | 0.006621059 | -1308.243 | -0.9535 |
| RAGTPYTVTLHGE | TNC | 0.006866234 | 888.161 | 0.697 |
| ILKETEFKKIKVL | EGFR | 0.007445671 | -903.569 | -0.9175 |
| GEVVVAEVGWDAL | TNC | 0.007588535 | 847.007 | 0.5785 |
| LFMRRRHIVRKRT | EGFR | 0.007926637 | -3424.9665 | -1.707 |
| ETVNLAWDNEMRV | TNC | 0.008115363 | 534.7985 | 0.374 |
| APSQIEVKDVTDT | TNC | 0.008284273 | 708.921 | 0.5435 |
| SDFLSNMSMDFQN | EGFR | 0.009048923 | -999.5805 | -0.809 |
| NKVEAAQNLTLPG | TNC | 0.009790343 | -1240.1265 | -1.0125 |
| GKVKALEEDNLSE | GLEA2 | 0.010438833 | 1041.3535 | 0.673 |
| NLKDPKNNILFKK | PHF3 | 0.011974617 | -904.3435 | -0.5075 |
| KSPQENLREPKRK | GLEA2 | 0.011985668 | -924.8655 | -0.778 |
| ALEEDNLSESSSE | GLEA2 | 0.01338913 | 1218.787 | 0.6815 |
| PKNNILFKKVLKG | PHF3 | 0.014111071 | -878.718 | -0.7685 |
| EGGVLKKVIRHKR | TNC | 0.014459747 | -1546.8 | -0.813 |
| YRALMDEEDMDDV | EGFR | 0.014666953 | 2564.201 | 0.764 |
| QIVFTHRINIPRR | TNC | 0.015208285 | -755.187 | -0.6435 |
| KFSDKEEHEQNDS | PHF3 | 0.015536046 | 1202.837 | 0.719 |
| ENLQIIRGNMYYE | EGFR | 0.0157327 | -1586.7025 | -0.6345 |
| NFYQVKDIRRPER | PHF3 | 0.016741913 | -1120.506 | -0.7945 |

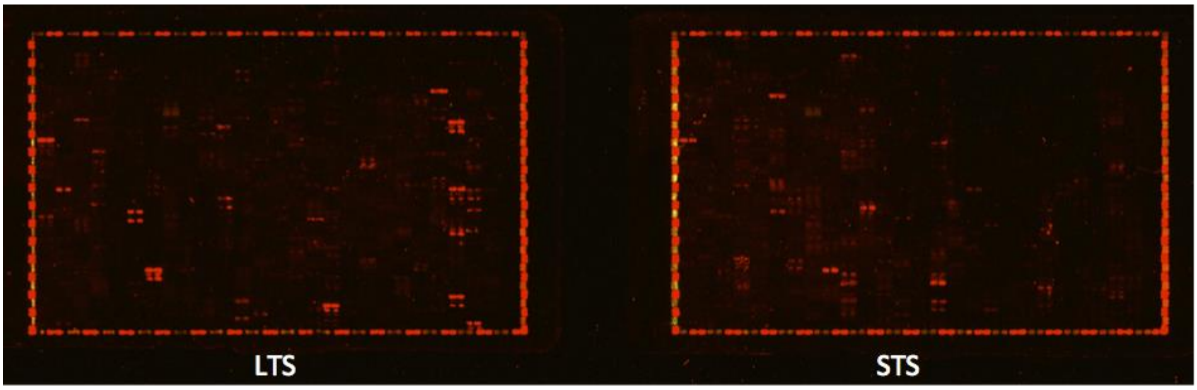


**Supplemental Figure 1:** Representative scans of screening PEPperCHIP® microarray (1745 peptides) for a long-term (LTS) and a short-term surviving (STS) patient. A red fluorescent labeled secondary antibody binding to the human heavy chain visualized patient antibodies specifically bound to spotted peptides on the array. The red spots on the border of the array denote control spots. Duplicate spots within this border depict antibodies against the candidate antigens.

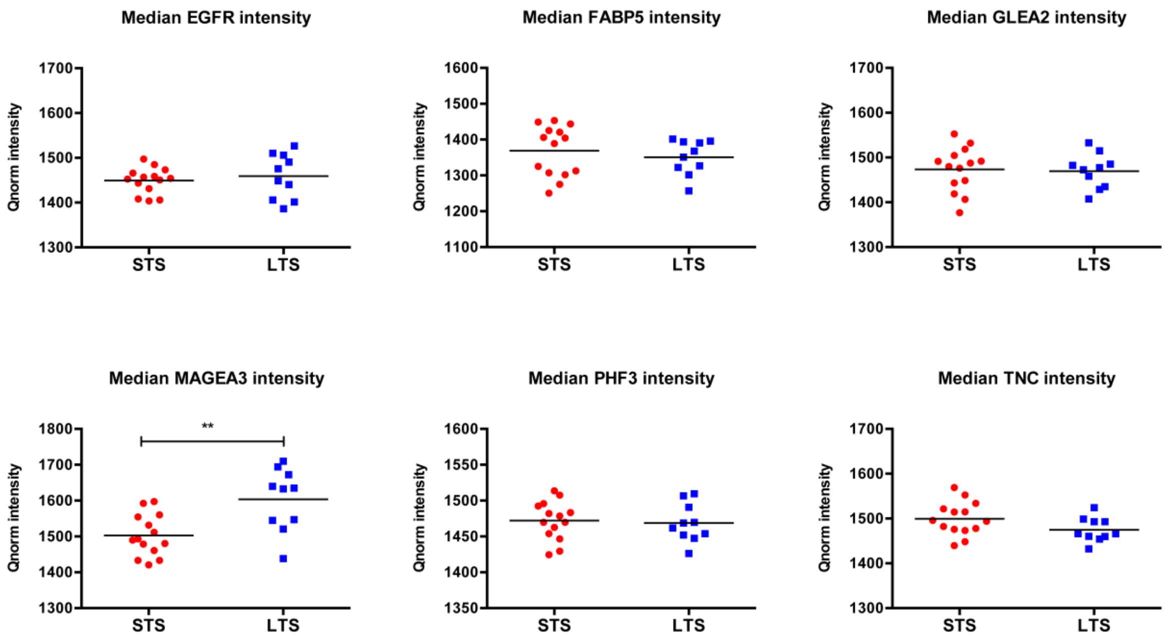


**Supplemental Figure 2 :** Median intensity for all peptides of an antigen on the 1745 PEPperCHIP® screening array. Every scatter plot illustrates the median centered, quantile normalized signal intensity for all peptides of an antigen per patient. Differences between STS and LTS patients are shown. P-values were calculated employing Student's t-test. *p<0.05, **p<0.01, ***p<0.001.

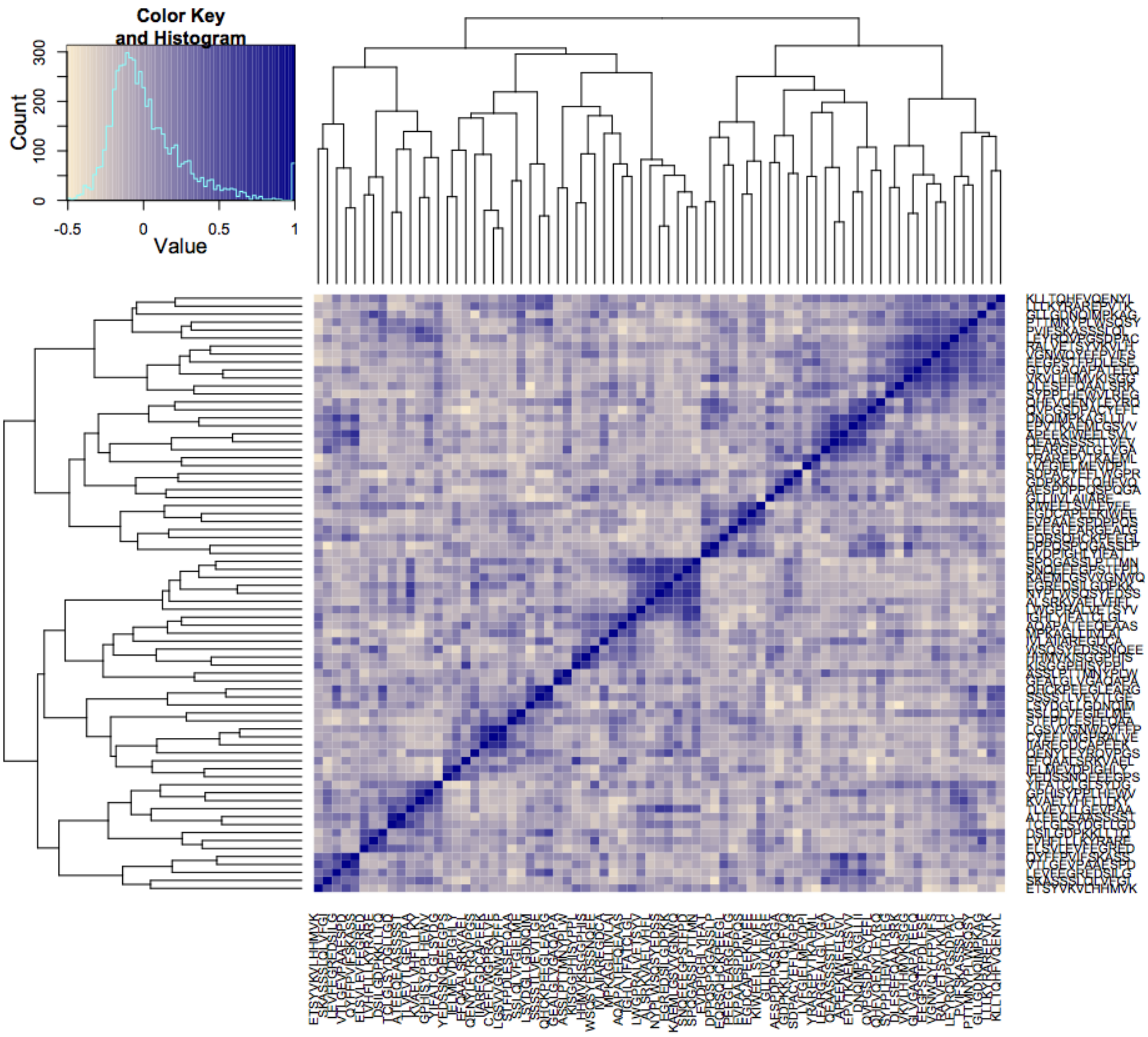


**Supplemental Figure 3 :** Correlation heatmap between MAGEA3 peptides on the PEPperCHIP® screening array. The signal of each MAGEA3 peptide across all patients is correlated with all other 74 MAGEA3 peptides on the screening array, clustered (hierarchical clustering) and illustrated in a heatmap. Blue color denotes a high correlation.

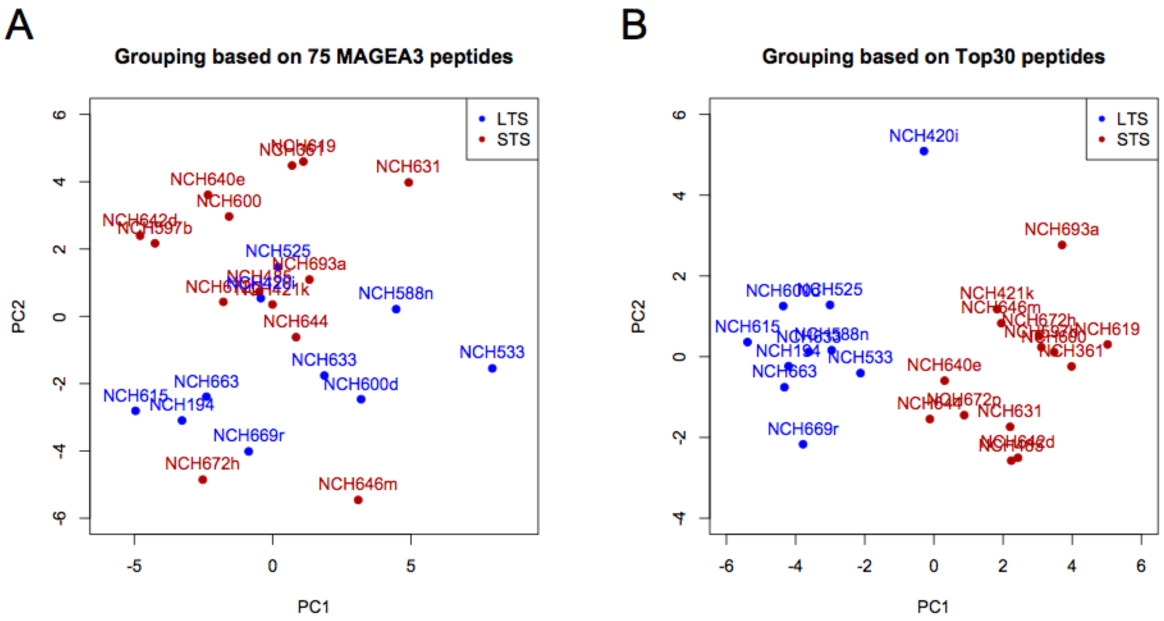


**Supplemental Figure 4.** Principal component analysis visualizing the grouping of LTS and STS patients according to the first and second principal component (PC1, PC2) for (A) all 75 MAGEA3 peptides on the screening array and (B) the Top30 peptides of the comparative analysis. Principal component analysis was performed on median centered, quantile normalized signal intensities of screening arrays.

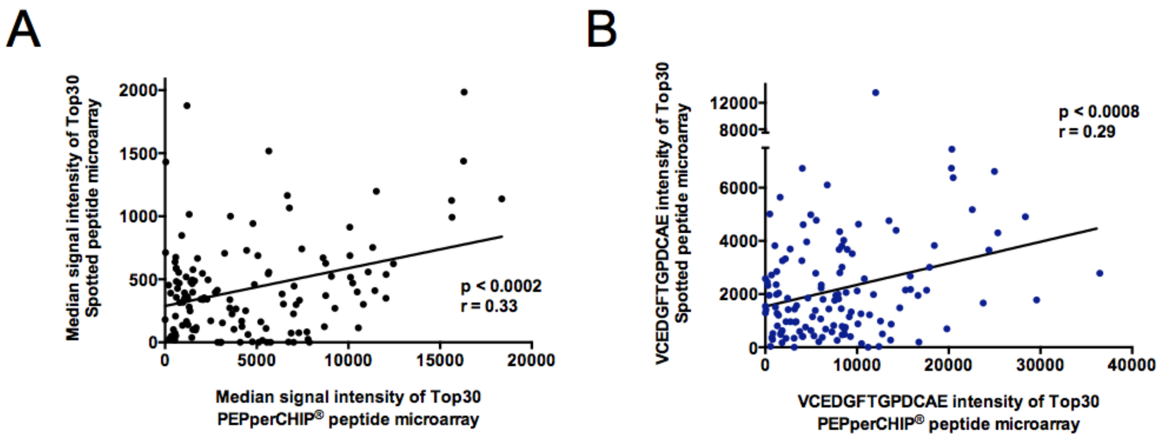


**Supplemental Figure 5**: Correlation between the signal intensities of the Top30 PEPperCHIP® peptide microarray and the Top30 spotted array (pre-synthesized peptides). Correlation of (A) median signal intensities and (B) VCEDGFTGPDCAE intensity for validation set 2 (n=129) are shown. P-values and Pearson's r were calculated using the Pearson correlation coefficient.

# Supplemental Material & Methods

**FASTA sequences of glioblastoma-associated antigens**

Top 30 peptides are color-coded in the FASTA sequence. Two pairs of PHF3 peptides were overlapping (yellow and red highlight). The prognostic anti-TNC peptide (VCEDGFTGPDCAE) is highlighted in green.

>sp|Q92576|PHF3_HUMAN **PHF3** OS=Homo sapiens GN=PHF3 PE=1 SV=3

MDIVDTFNHLIPTEHLDDALFLGSNLENEVCEDFSASQNVLEDSLKNMLSDKDPMLGSASN
QFCLPVLDSNDPNFQMPCSTVVGLDDIMDEGVVKESGNDTIDEEELILPNRNLRDKVEENS
VRSPRKSPRLMAQEQVRSLRQSTIAKRSNAAPLSNTKKASGKTVSTAKAGVKQPERSQVK
EEVCMSLKPEYHKENRRCSRNSGQIEVVPEVSVSSSHSSVSSCLEMKDEDGLDSKHKCNN
PGEIDVPSHELNCSLLSETCVTIGEKKNEALMECKAKPVGSPLFKFSDKEEHEQNDSISGKT
GETVVEEMIATRKVEQDSKETVKLSHEDDHILEDAGSSDISSDAACTNPNKTENSLVGLPSC
VDEVTECNLELKDTMGIADKTENTLERNKIEPLGYCEDAESNRQLESTEFNKSNLEVVDTST
FGPESNILENAICDVPDQNSKQLNAIESTKIESHETANLQDDRNSQSSSVSYLESKSVKSKH
TKPVIHSKQNMTTDAPKKIVAAKYEVIHSKTKVNVKSVKRNTDVPESQQNFHRPVKVRKKQI
DKEPKIQSCNSGVKSVKNQAHSVLKKTLQDQTLVQIFKPLTHSLSDKSHAHPGCLKEPHHP
AQTGHVSHSSQKQCHKPQQQAPAMKTNSHVKEELEHPGVEHFKEEDKLKLKKPEKNLQP
RQRRSSKSFSLDEPPLFIPDNIATIRREGSDHSSSFESKYMWTPSKQCGFCKKPHGNRFMV
GCGRCDDWFHGDCVGLSLSQAQQMGEEDKEYVCVKCCAEEDKKTEILDPDTLENQATVE
FHSGDKTMECEKLGLSKHTTNDRTKYIDDTVKHKVKILKRESGEGRNSSDCRDNEIKKWQL
APLRKMGQPVLPRRSSEEKSEKIPKESTTVTCTGEKASKPGTHEKQEMKKKKVEKGVLNVH
PAASASKPSADQIRQSVRHSLKDILMKRLTDSNLKVPEEKAAKVATKIEKELFSFFRDTDAKY
KNKYRSLMFNLKDPKNNILFKKVLKGEVTPDHLIRMSPEELASKELAAWRRRENRHTIEMIE
KEQREVERRPITKITHKGEIEIESDAPMKEQEAAMEIQEPAANKSLEKPEGSEKQKEEVDSM
SKDTTSQHRQHLFDLNCKICIGRMAPPVDDLSPKKVKVVVGVARKHSDNEAESIADALSSTS
NILASEFFEEEKQESPKSTFSPAPRPEMPGTVEVESTFLARLNFIWKGFINMPSVAKFVTKA
YPVSGSPEYLTEDLPDSIQVGGRISPQTVWDYVEKIKASGTKEICVVRFTPVTEEDQISYTLL
FAYFSSRKRYGVAANNMKQVKDMYLIPLGATDKIPHPLVPFDGPGLELHRPNLLLGLIIRQKL
KRQHSACASTSHIAETPESAPPIALPPDKKSKIEVSTEEAPEEENDFFNSFTTVLHKQRNKP
QQNLQEDLPTAVEPLMEVTKQEPPKPLRFLPGVLIGWENQPTTLELANKPLPVDDILQSLLG
TTGQVYDQAQSVMEQNTVKEIPFLNEQTNSKIEKTDNVEVTDGENKEIKVKVDNISESTDKS
AEIETSVVGSSSISAGSLTSLSLRGKPPDVSTEAFLTNLSIQSKQEETVESKEKTLKRQLQED
QENNLQDNQTSNSSPCRSNVGKGNIDGNVSCSENLVANTARSPQFINLKRDPRQAAGRSQ
PVTTSESKDGDSCRNGEKHMLPGLSHNKEHLTEQINVEEKLCSAEKNSCVQQSDNLKVAQ
NSPSVENIQTSQAEQAKPLQEDILMQNIETVHPFRRGSAVATSHFEVGNTCPSEFPSKSITF
TSRSTSPRTSTNFSPMRPQQPNLQHLKSSPPGFPFPGPPNFPPQSMFGFPPHLPPPLLPP
PGFGFAQNPMVPWPPVVHLPGQPQRMMGPLSQASRYIGPQNFYQVKDIRRPERRHSDP
WGRQDQQQLDRPFNRGKGDRQRFYSDSHHLKRERHEKEWEQESERHRRRDRSQDKDR
DRKSREEGHKDKERARLSHGDRGTDGKASRDSRNVDKKPDKPKSEDYEKDKEREKSKHR
EGEKDRDRYHKDRDHTDRTKSKR

>sp|Q9BVI0|PHF20_HUMAN **GLEA2** OS=Homo sapiens GN=PHF20 PE=1 SV=2

MTKHPPNRRGISFEVGAQLEARDRLKNWYPAHIEDIDYEEGKVLIHFKRWNHRYDEWFCW
DSPYLRPLEKIQLRKEGLHEEDGSSEFQINEQVLACWSDCRFYPAKVTAVNKDGTYTVKFY
DGVVQTVKHIHVKAFSKDQNIVGNARPKETDHKSLSSSPDKREKFKEQRKATVNVKKDKED
KPLKTEKRPKQPDKEGKLICSEKGKVSEKSLPKNEKEDKENISENDREYSGDAQVDKKPEN
DIVKSPQENLREPKRKRGRPPSIAPTAVDSNSQTLQPITLELRRRKISKGCEVPLKRPRLDKN
SSQEKSKNYSENTDKDLSRRRSSRLSTNGTHEILDPDLVVSDLVDTDPLQDTLSSTKESEE

GQLKSALEAGQVSSALTCHSFGDGSGAAGLELNCPSMGENTMKTEPTSPLVELQEISTVEV
TNTFKKTDDFGSSNAPAVDLDHKFRCKVVDCLKFFRKAKLLHYHMKYFHGMEKSLEPEESP
GKRHVQTRGPSASDKPSQETLTRKRVSASSPTTKDKEKNKEKKFKEFVRVKPKKKKKKKK
KTKPECPCSEEISDTSQEPSPPKAFAVTRCGSSHKPGVHMSPQLHGPESGHHKGKVKALE
EDNLSESSSESFLWSDDEYGQDVDVTTNPDEELDGDDRYDFEVVRCICEVQEENDFMIQC
EECQCWQHGVCMGLLEENVPEKYTCYVCQDPPGQRPGFKYWYDKEWLSRGHMHGLAFL
EENYSHQNAKKIVATHQLLGDVQRVIEVLHGLQLKMSILQSREHPDLPLWCQPWKQHSGE
GRSHFRNIPVTDTRSKEEAPSYRTLNGAVEKPRPLALPLPRSVEESYITSEHCYQKPRAYYP
AVEQKLVVETRGSALDDAVNPLHENGDDSLSPRLGWPLDQDRSKGDSDPKPGSPKVKEY
VSKKALPEEAPARKLLDRGGEGLLSSQHQWQFNLLTHVESLQDEVTHRMDSIEKELDVLES
WLDYTGELEPPEPLARLPQLKHCIKQLLMDLGKVQQIALCCST

>sp|P24821|TENA_HUMAN **Tenascin C** OS=Homo sapiens GN=TNC PE=1 SV=3

MGAMTQLLAGVFLAFLALATEGGVLKKVIRHKRQSGVNATLPEENQPVVFNHVYNIKLPVG
SQCSVDLESASGEKDLAPPSEPSESFQEHTVDGENQIVFTHRINIPRRACGCAAAPDVKELL
SRLEELENLVSSLREQCTAGAGCCLQPATGRLDTRPFCSGRGNFSTEGCGCVCEPGWKG
PNCSEPECPGNCHLRGRCIDGQCICDDGFTGEDCSQLACPSDCNDQGKCVNGVCICFEGY
AGADCSREICPVPCSEEHGTCVDGLCVCHDGFAGDDCNKPLCLNNCYNRGRCVENECVC
DEGFTGEDCSELICPNDCFDRGRCINGTCYCEEGFTGEDCGKPTCPHACHTQGRCEEGQ
CVCDEGFAGVDCSEKRCPADCHNRGRCVDGRCECDDGFTGADCGELKCPNGCSGHGRC
VNGQCVCDEGYTGEDCSQLRCPNDCHSRGRCVEGKCVCEQGFKGYDCSDMSCPNDCH
QHGRCVNGMCVCDDGYTGEDCRDRQCPRDCSNRGLCVDGQCVCEDGFTGPDCAELSC
PNDCHGQGRCVNGQCVCHEGFMGKDCKEQRCPSDCHGQGRCVDGQCICHEGFTGLDC
GQHSCPSDCNNLGQCVSGRCICNEGYSGEDCSEVSPPKDLVVTEVTEETVNLAWDNEMR
VTEYLVVYTPTHEGGLEMQFRVPGDQTSTIIQELEPGVEYFIRVFAILENKKSIPVSARVATYL
PAPEGLKFKSIKETSVEVEWDPLDIAFETWEIIFRNMNKEDEGEITKSLRRPETSYRQTGLAP
GQEYEISLHIVKNNTRGPGLKRVTTTRLDAPSQIEVKDVTDTTALITWFKPLAEIDGIELTYGIK
DVPGDRTTIDLTEDENQYSIGNLKPDTEYEVSLISRRGDMSSNPAKETFTTGLDAPRNLRRV
SQTDNSITLEWRNGKAAIDSYRIKYAPISGGDHAEVDVPKSQQATTKTTLTGLRPGTEYGIG
VSAVKEDKESNPATINAATELDTPKDLQVSETAETSLTLLWKTPLAKFDRYRLNYSLPTGQW
VGVQLPRNTTSYVLRGLEPGQEYNVLLTAEKGRHKSKPARVKASTEQAPELENLTVTEVG
WDGLRLNWTAADQAYEHFIIQVQEANKVEAARNLTVPGSLRAVDIPGLKAATPYTVSIYGVI
QGYRTPVLSAEASTGETPNLGEVVVAEVGWDALKLNWTAPEGAYEYFFIQVQEADTVEAA
QNLTVPGGLRSTDLPGLKAATHYTITIRGVTQDFSTTPLSVEVLTEEVPDMGNLTVTEVSWD
ALRLNWTTPDGTYDQFTIQVQEADQVEEAHNLTVPGSLRSMEIPGLRAGTPYTVTLHGEVR
GHSTRPLAVEVVTEDLPQLGDLAVSEVGWDGLRLNWTAADNAYEHFVIQVQEVNKVEAAQ
NLTLPGSLRAVDIPGLEAATPYRVSIYGVIRGYRTPVLSAEASTAKEPEIGNLNVSDITPESFN
LSWMATDGIFETFTIEIIDSNRLLETVEYNISGAERTAHISGLPPSTDFIVYLSGLAPSIRTKTIS
ATATTEALPLLENLTISDINPYGFTVSWMASENAFDSFLVTVVDSGKLLDPQEFTLSGTQRKL
ELRGLITGIGYEVMVSGFTQGHQTKPLRAEIVTEAEPEVDNLLVSDATPDGFRLSWTADEGV
FDNFVLKIRDTKKQSEPLEITLLAPERTRDITGLREATEYEIELYGISKGRRSQTVSAIATTAM
GSPKEVIFSDITENSATVSWRAPTAQVESFRITYVPITGGTPSMVTVDGTKTQTRLVKLIPGV
EYLVSIIAMKGFEESEPVSGSFTTALDGPSGLVTANITDSEALARWQPAIATVDSYVISYTGE
KVPEITRTVSGNTVEYALTDLEPATEYTLRIFAEKGPQKSSTITAKFTTDLDSPRDLTATEVQS
ETALLTWRPPRASVTGYLLVYESVDGTVKEVIVGPDTTSYSLADLSPSTHYTAKIQALNGPL
RSNMIQTIFTTIGLLYPFPKDCSQAMLNGDTTSGLYTIYLNGDKAEALEVFCDMTSDGGGWI
VFLRRKNGRENFYQNWKAYAAGFGDRREEFWLGLDNLNKITAQGQYELRVDLRDHGETAF
AVYDKFSVGDAKTRYKLKVEGYSGTAGDSMAYHNGRSFSTFDKDTDSAITNCALSYKGAF
WYRNCHRVNLMGRYGDNNHSQGVNWFHWKGHEHSIQFAEMKLRPSNFRNLEGRRKRA

>sp|Q01469|**FABP5_**HUMAN Fatty acid-binding protein, epidermal OS=Homo sapiens GN=FABP5 PE=1 SV=3

MATVQQLEGRWRLVDSKGFDEYMKELGVGIALRKMGAMAKPDCIITCDGKNLTIKTESTLK
TTQFSCTLGEKFEETTADGRKTQTVCNFTDGALVQHQEWDGKESTITRKLKDGKLVVECV
MNNVTCTRIYEKVE

>sp|P43357|MAGA3_HUMAN **MAGEA3** OS=Homo sapiens GN=MAGEA3 PE=1 SV=1

MPLEQRSQHCKPEEGLEARGEALGLVGAQAPATEEQEAASSSSTLVEVTLGEVPAAESPD
PPQSPQGASSLPTTMNYPLWSQSYEDSSNQEEEGPSTFPDLESEFQAALSRKVAELVHFL
LLKYRAREPVTKAEMLGSVVGNWQYFFPVIFSKASSSLQLVFGIELMEVDPIGHLYIFATCLG
LSYDGLLGDNQIMPKAGLLIIVLAIIAREGDCAPEEKIWEELSVLEVFEGREDSILGDPKKLLT
QHFVQENYLEYRQVPGSDPACYEFLWGPRALVETSYVKVLHHMVKISGGPHISYPPLHEW
VLREGEE

>sp|P00533|EGFR_HUMAN **EGFR** OS=Homo sapiens GN=EGFR PE=1 SV=2

MRPSGTAGAALLALLAALCPASRALEEKKVCQGTSNKLTQLGTFEDHFLSLQRMFNNCEVV
LGNLEITYVQRNYDLSFLKTIQEVAGYVLIALNTVERIPLENLQIIRGNMYYENSYALAVLSNY
DANKTGLKELPMRNLQEILHGAVRFSNNPALCNVESIQWRDIVSSDFLSNMSMDFQNHLGS
CQKCDPSCPNGSCWGAGEENCQKLTKIICAQQCSGRCRGKSPSDCCHNQCAAGCTGPR
ESDCLVCRKFRDEATCKDTCPPLMLYNPTTYQMDVNPEGKYSFGATCVKKCPRNYVVTDH
GSCVRACGADSYEMEEDGVRKCKKCEGPCRKVCNGIGIGEFKDSLSINATNIKHFKNCTSIS
GDLHILPVAFRGDSFTHTPPLDPQELDILKTVKEITGFLLIQAWPENRTDLHAFENLEIIRGRT
KQHGQFSLAVVSLNITSLGLRSLKEISDGDVIISGNKNLCYANTINWKKLFGTSGQKTKIISNR
GENSCKATGQVCHALCSPEGCWGPEPRDCVSCRNVSRGRECVDKCNLLEGEPREFVENS
ECIQCHPECLPQAMNITCTGRGPDNCIQCAHYIDGPHCVKTCPAGVMGENNTLVWKYADA
GHVCHLCHPNCTYGCTGPGLEGCPTNGPKIPSIATGMVGALLLLLVVALGIGLFMRRRHIVR
KRTLRRLLQERELVEPLTPSGEAPNQALLRILKETEFKKIKVLGSGAFGTVYKGLWIPEGEKV
KIPVAIKELREATSPKANKEILDEAYVMASVDNPHVCRLLGICLTSTVQLITQLMPFGCLLDYV
REHKDNIGSQYLLNWCVQIAKGMNYLEDRRLVHRDLAARNVLVKTPQHVKITDFGLAKLLG
AEEKEYHAEGGKVPIKWMALESILHRIYTHQSDVWSYGVTVWELMTFGSKPYDGIPASEISS
ILEKGERLPQPPICTIDVYMIMVKCWMIDADSRPKFRELIIEFSKMARDPQRYLVIQGDERMH
LPSPTDSNFYRALMDEEDMDDVVDADEYLIPQQGFFSSPSTSRTPLLSSLSATSNNSTVACI
DRNGLQSCPIKEDSFLQRYSSDPTGALTEDSIDDTFLPVPEYINQSVPKRPAGSVQNPVYHN
QPLNPAPSRDPHYQDPHSTAVGNPEYLNTVQPTCVNSTFDSPAHWAQKGSHQISLDNPDY
QQDFFPKEAKPNGIFKGSTAENAEYLRVAPQSSEFIGA
